# Elevated Serum Interleukin-23 Levels in Patients with Oral and Cutaneous Lichen Planus

**DOI:** 10.1155/2021/5578568

**Published:** 2021-07-12

**Authors:** Maryam Mardani, Hossein Mofidi, Ladan Dastgheib, Sara Ranjbar, Nasrin Hamidizadeh

**Affiliations:** ^1^Oral and Dental Disease Research Center, School of Dentistry, Shiraz University of Medical Sciences, Shiraz, Iran; ^2^Molecular Dermatology Research Center, Shiraz University of Medical Sciences, Shiraz, Iran

## Abstract

Lichen planus is considered a chronic inflammatory disease which affects different sites, such as the skin, mucous membranes, hair, and nails. Based on the evidence, a complex cytokine network plays a crucial role in lichen planus pathogenesis. The study was aimed at assessing the serum IL-23 levels in the patients with cutaneous and oral lichen planus compared to healthy controls. *Method*. The study included 30 cutaneous lichen planus patients, 20 oral lichen planus patients, and 33 control subjects. Five milliliters of peripheral blood was obtained from each patient, and the serum was separated. IL-23 levels were determined using the ELISA kit, and the data were analyzed using the Mann–Whitney test. *Results*. IL-23 levels in the patient serum with oral lichen planus (*P* value ≤ 0.001) were significantly higher than in controls. Furthermore, there were significant differences in IL-23 serum levels in the patients with cutaneous lichen planus compared to the healthy controls (*P* value ≤ 0.001). Moreover, IL-23 serum levels were statistically different between patients with cutaneous lichen planus and patients with oral lichen planus (*P* value ≤ 0.001). Based on the mean concentration of interleukin-23, IL-23 levels were higher in the patients with oral lichen planus than in the patients with cutaneous lichen planus. *Conclusions*. Elevated serum IL-23 levels in the patients with oral lichen planus may indicate that IL-23 plays a crucial role in the pathogenesis of oral lichen planus. However, more research is needed with a larger sample size.

## 1. Introduction

Lichen planus (LP) is a chronic inflammatory disease which affects various parts of the body such as the skin, mucous membranes, hair, and nails. LP is common in 0.5-2% of the world population and has a variety of clinical subtypes based on the morphology of the lesions and the location of involvement [1, 2]. Oral lichen planus is more common and could affect the general population by 0.5% to 4% [3, 4]. OLP is characterized by a subepithelial infiltration of T lymphocytes and the degeneration of basal keratinocytes [5–7].

The dysregulation of inflammatory cytokines is the cause of various autoimmune diseases and allergies. IL-23 is a heterodimeric cytokine which belongs to the IL-12 family which contains a p40 and a unique p19 subunit. IL-23 is mainly produced by the dendritic cells and macrophages and secreted by various cell types, such as activated dendritic cells, macrophages, and epithelia, and acts as an essential driving factor for the immune response [8]. IL-23 binds to CD4 + T cells through the IL-23R and contributes to the maintenance and induction of TH17 to produce various cytokines, including IL-17A (IL-17), IL-17F, IL-22, IL-26, IFN-g, CCL20, and TNF-*α*; IL-17 activates the production of numerous inflammatory molecules such as cytokines, chemokines, defensins, and MMPs by activating the epithelia, endothelia, fibroblasts, chondrocytes, and osteoblasts [9, 10].

IL-23 is believed to be the main cytokine in the pathogenesis of inflammatory and autoimmune diseases [11, 12]. Furthermore, the evidence shows that the IL-23/IL-17 axis plays a critical role in the severity and chronic course of rheumatoid arthritis [13]. Besides, some previous research reported IL-23 involvement in the autoimmune diseases, such as psoriasis and rheumatoid arthritis [14]. Immune-inflammatory factors and cytokines were thought to play critical roles in the development and immunopathogenesis of OLP [15]. Regarding the pathogenic role of cytokines in LP diseases, TNF-*α*, IFN*γ*, IL-10, IL-17, and IL-22 are to be involved in the pathogenesis of OLP [15]. IL-6, IL-10, INF-*α*, and TNF-*α* cytokines are also involved in the LP pathogenesis [16]. TNF-*α* production in saliva associated to OLP activity was also increased in the OLP patients [17]. The IL-17 serum level was higher in the patients with OLP compared to controls [18]. An increased serum concentration of IL-17 and a high expression in a skin lesion was reported in another study [19].

Few studies examined the IL-23 and its pathogenic role in LP. The present study was aimed at determining the IL-23 serum level in OLP and CLP patients compared to the controls.

## 2. Materials and Method

In this cross-sectional study, the entire sample comprised 50 patients: 30 patients with CLP and 20 patients with OLP, who were referred to the Oral Medicine Department and Molecular Dermatology Research Center, Shiraz University of Medical Sciences, Shiraz, Iran. 33 healthy control persons took part in this study.

Patients were matched with controls on age and gender. LP was confirmed both clinically and histopathologically. All selected participants did not use any systemic and topical drugs related to a leukemic reaction in the past three months and did not receive chemotherapy or radiation therapy in the past three months. Moreover, they had no inflammatory and autoimmune diseases or any cancer and were all nonsmokers. All participants have signed the consent form. Ethical approval for this study was approved by Shiraz University of Medical Sciences, Shiraz, Iran.

### 2.1. Collection of Samples

Approximately 5 ml of peripheral blood was obtained from each subject. The samples were centrifuged to separate the serum and then stored at -20°C until further analysis.

### 2.2. Cytokine Assay

The IL-23 enzyme-linked immunosorbent assay (ELISA) kit (MyBioSource, San Diego, California, USA) was used to detect the IL-23 levels in the serum. Results were expressed in pg/ml for IL-23 in the serum.

### 2.3. Statistical Analysis

SPSS version 18 (IBM) was used for the statistical analysis, and the obtained results were expressed as the mean ± standard deviation (SD). The Mann–Whitney test was performed to indicate the difference in IL-23 levels between patients and controls.

## 3. Results

The serum interleukin-23 levels were assessed in 30 CLP, 20 OLP, and 33 controls. The baseline characteristics of LP patients and controls are listed in Table 1.

**Figure 1:**
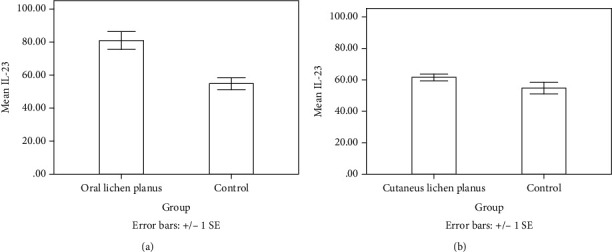
Comparison of the mean IL-23 serum levels between healthy controls and LP patients: (a) oral lichen planus (*P* value ≤ 0.001).); (b) cutaneous lichen planus (*P* value ≤ 0.001).

The IL-23 concentration in the serum of patients with LP and healthy controls is shown in Table 2. As Table 3 shows, the serum levels of IL-23 in OLP patients were significantly higher than those in the controls (*P* value ≤ 0.001). Serum IL-23 levels were also higher in the patients with CLP than in the control group (*P* value ≤ 0.001). The mean serum IL-23 level was also statistically higher in the patients with OLP than in the patients with CLP (*P* value ≤ 0.001) (Figure 1).

## 4. Discussion

Th17 and IL-23/IL-17 signaling pathways are implicated in many autoimmune diseases.

In this regard, previous research showed that the increased levels of IL-23 and IL-17 are linked to the pathogenesis of many autoimmune diseases, such as pediatric systemic lupus erythematosus [20], systemic lupus erythematosus, multiple sclerosis ankylosing spondylitis, Graves' disease, Crohn's disease [21] psoriasis, psoriasis arthritis [22, 23], chronic spontaneous urticarial [24], morphea [25], bullous pemphigoid [26], pemphigus vulgaris [27, 28], pemphigus foliaceus [29], and vitiligo [30].

Several studies have looked at the serum cytokine levels and saliva of patients with LP. However, concerning these controversial results, none of the cytokines studied suggests being the most useful indicator of the disease.

Xie et al. found a significantly elevated proportion of Th1 and Th17 cells in the peripheral blood and a significant increase in IL-17 serum levels in OLP patients, which might play an important role in the OLP pathogenesis [18].

In this regard, a published report showed elevated IL-17 and IL-23 serum levels in OLP patients with chronic periodontitis compared to healthy controls [31]. Significantly higher IL-23 serum levels were also observed in the patients with CLP and combined CLP and OLP patients than in healthy controls [32].

Our results showed an increased IL-23 level in the serum of CLP and OLP patients compared to controls. Besides, serum IL-23 levels in the OLP patients were significantly higher than serum IL-23 levels in the patients with CLP. Chen et al. found the same results for LP lesions. They showed increased expression of IL-22 and IL-23 in LP lesions and overexpression of IL-22 and IL-23 in OLP than in CLP [33, 34].

The role of the IL-23/IL-17 axis in the autoimmune and inflammatory diseases was commonly described. It appears that the IL-23/IL-17 axis also plays an important role in OLP pathogenesis.

In this regard, Lu et al. indicated the involvement of the IL-23/IL-17 axis in OLP pathogenesis. They suggested an interaction between T cells and the keratinocytes, in which the keratinocytes produce IL-23 in OLP lesions. Then, the IL-23 produced leads to the accumulation of Th17 cells and consequently to IL-17 overproduction in the local lesions of OLP. On the other hand, IL-17 induces the keratinocytes to produce various inflammatory mediators and form a complex immune network near OLP lesions [35].

Despite the advances in deciphering the Th17 transcription network, we still lack a clear understanding of the IL-23-dependent mechanisms which control the pathogenic Th17 differentiation process.

Therefore, STAT3-activated IL-23 is indispensable for the mediation of autoimmune pathology through the binding to pathogenic TH17 cells. The IL-23-activated Th17 cells promote chronic tissue inflammation, granuloma formation, and autoimmunity. Accumulated data clearly show that TGF*β*, IL-6, and IL-1*β* are essential elements that initiate Th17 cell development, while exposure to IL-23 is necessary for the differentiation and maturation of inflammatory Th17 cells. The ability of IL-23 to differentiate and stabilize pathogenic Th17 cells takes place through IL-23R expression upregulation, which in turn is STAT3-dependent. Moreover, TGF*β*3 is induced by IL-23 and improves IL-23R expression.

Hence, IL-23 induces IFN*γ* expression in Th17 cells, and IFN*γ* + IL-17 + cells are highly pathogenic. Finally, it is important to mention that the skin resident cells, such as keratinocytes, fibroblasts, and endothelial cells, respond to IL-17. It is known that the STAT3-activated IL-23 is indispensable for mediating autoimmune pathology through the binding to pathogenic TH17 cells. The IL-23-activated Th17 cells promote chronic tissue inflammation, granuloma formation, and autoimmunity [36]. The accumulated data clearly shows that TGF*β*, IL-6, and IL-1*β* are essential elements which initiate the development of Th17 cells, while exposure to IL-23 is necessary for the differentiation and maturation of inflammatory Th17 cells. The ability of IL-23 to differentiate and stabilize pathogenic Th17 cells is due to the upregulation of IL-23R expression which in turn is STAT3-dependent. Furthermore, TGF*β*3 is induced by IL-23 and improves IL-23R expression. IL-23 induces the IFN*γ* expression in Th17 cells, and IFN*γ* + IL-17 + cells are highly pathogenic. Finally, the skin-resident cells such as keratinocytes, fibroblasts, and endothelial cells react to IL-17 [36].

A better understanding of the IL-23/IL-17 axis role in LP pathogenesis could help develop new therapeutic strategies for the prevention and management of LP in the future.

Thus, the drugs which target the IL-23/IL-17 axis may also result in the improved efficacy to treat the LP. In particular, when both IL-23 and IL-17 were blocked, remarkable results were obtained. The effectiveness of such drugs to treat psoriasis and psoriatic arthritis was documented [22, 23].

Monoclonal antibodies against IL-23 alone or in combination with IL-12 were shown to be an effective therapy for psoriasis, multiple sclerosis, and systemic lupus erythematosus [37–43]. These antibodies may also be effective to treat the LP.

## 5. Conclusions

It appears that elevated serum IL-23 levels may be associated to its pathogenic role in LP, particularly OLP. However, more research with a larger sample size is required.

## Figures and Tables

**Table 1:** Baseline characteristics of the study population with OLP, CLP, and controls.

Diagnosis	Cases (*n*)	Mean ± SD age (years)	Age range (years)	Females (*n*)	Males (*n*)
Oral LP	20	51.31 ± 13.68	28~84	16	4
Cutaneous LP	30	43.53 ± 15.95	17~72	20	10
Control	33	48.68 ± 9.95	17~65	26	7

**Table 2:** IL-23 concentration in patients with OLP and CLP and in controls.

ID/participants	IL-23 (pg/ml) OLP patients	IL-23 (pg/ml) CLP patients	IL-23 (pg/ml) Controls
1	93.38	56.08	84.51
2	67.09	56.08	48.28
3	58.37	60.21	19.03
4	72.14	60.66	42.31
5	81.55	58.37	145.15
6	148.11	81.55	45.98
7	56.37	55.82	41.39
8	73.05	59.23	45.38
9	120.99	50.11	45.52
10	116.56	58.37	53.78
11	97.33	56.08	42.31
12	82.04	74.89	51.95
13	82.03	50.11	92.40
14	68.42	50.57	45.52
15	55.16	89.44	54.70
16	65.25	92.89	38.68
17	69.84	51.49	41.39
18	67.55	57.91	53.78
19	61.58	52.41	61.58
20	83.03	68.92	48.28
21		60.21	45.98
22		76.72	47.82
23		48.28	50.72
24		68.01	41.39
25		62.96	68.46
26		57.45	51.03
27		63.88	54.70
28		60.66	51.95
29		57.45	56.54
30		48.74	56.54
31			56.08
32			53.78
33			68.92

**Table 3:** Comparison of IL-23 levels in patients with OLP and CLP and control groups.

Variable	IL − 23 (pg/ml) ± STD	Std. error	*n*	*P* value
Oral LP	80.99 ± 24.07	5.38	20	≤0.001
Control	54.72 ± 20.83	3.62	33	
Cutaneous LP	61.52 ± 11.42	2.08	30	≤0.001
Control	54.72 ± 20.83	3.62	33	
Cutaneous LP	61.52 ± 11.42	2.08	30	≤0.001
Oral LP	80.99 ± 24.07	5.38	20	

## Data Availability

Data are found in the supplementary information files.

## References

[1] Gorouhi F., Davari P., Fazel N. (2014). Cutaneous and mucosal lichen planus: a comprehensive review of clinical subtypes, risk factors, diagnosis, and prognosis. *Scientific World Journal*.

[2] Mollaoglu N. (2000). Oral lichen planus: a review. *The British Journal of Oral & Maxillofacial Surgery*.

[3] Gupta S., Jawanda M. K. (2015). Oral lichen planus: an update on etiology, pathogenesis, clinical presentation, diagnosis and management. *Indian Journal of Dermatology*.

[4] Nico M. M., Fernandes J. D., Lourenço S. V. (2011). Oral lichen planus. *Anais Brasileiros de Dermatologia*.

[5] Lavanya N., Rao U. K., Jayanthi P., Ranganathan K. (2011). Oral lichen planus: an update on pathogenesis and treatment. *Journal of Oral and Maxillofacial Pathology*.

[6] Sugerman P., Savage N. W., Walsh L. J. (2002). The pathogenesis of oral lichen planus. *Critical Reviews in Oral Biology & Medicine*.

[7] Roopashree M., Gondhalekar R. V., Shashikanth M. C., George J., Thippeswamy S. H., Shukla A. (2010). Pathogenesis of oral lichen planus–a review. *Journal of Oral Pathology & Medicine*.

[8] Tan Z. Y., Bealgey K. W., Fang Y., Gong Y. M., Bao S. (2009). Interleukin-23: immunological roles and clinical implications. *The International Journal of Biochemistry & Cell Biology*.

[9] Wilson N. J., Boniface K., Chan J. R. (2007). Development, cytokine profile and function of human interleukin 17-producing helper T cells. *Nature Immunology*.

[10] Parham C., Chirica M., Timans J. (2002). A receptor for the heterodimeric cytokine IL-23 is composed of IL-12R*β*1 and a novel cytokine receptor subunit, IL-23R. *The Journal of Immunology*.

[11] Croxford A. L., Mair F., Becher B. (2012). IL-23: one cytokine in control of autoimmunity. *European Journal of Immunology*.

[12] Tang C., Chen S., Qian H., Huang W. (2012). Interleukin-23: as a drug target for autoimmune inflammatory diseases. *Immunology*.

[13] Lubberts E. (2015). The IL-23-IL-17 axis in inflammatory arthritis. *Nature Reviews Rheumatology*.

[14] Yuan N., Yu G., Liu D., Wang X., Zhao L. (2019). An emerging role of interleukin-23 in rheumatoid arthritis. *Immunopharmacology and Immunotoxicology*.

[15] Ma H., Wu Y., Yang H. (2016). MicroRNAs in oral lichen planus and potential miRNA-mRNA pathogenesis with essential cytokines: a review. *Oral Surgery, Oral Medicine, Oral Pathology and Oral Radiology*.

[16] Agha-Hosseini F., Moosavi M.-S., Tabrizi M. H. (2015). Comparison of oral lichen planus and systemic lupus erythematosus in interleukins level. *Archives of Iranian Medicine (AIM)*.

[17] Zahran F., Shaker O., Ghalwash D., Fahmy M., Mostafa M., Al-Attas S. (2015). Salivary TNF-[alpha] and sCD44 as markers for disease activity and malignant transformation in oral lichen planus. *Advances in Environmental Biology*.

[18] Xie S., Ding L., Xiong Z., Zhu S. (2012). Implications of Th1 and Th17 cells in pathogenesis of oral lichen planus. *Journal of Huazhong University of Science and Technology. Medical Sciences*.

[19] Żychowska M., Batycka-Baran A., Baran W. (2020). Increased serum level and high tissue immunoexpression of interleukin 17 in cutaneous lichen planus: a novel therapeutic target for recalcitrant cases?. *Disease Markers*.

[20] Rana A., Minz R. W., Aggarwal R., Anand S., Pasricha N., Singh S. (2012). Gene expression of cytokines (TNF-*α*, IFN-*γ*), serum profiles of IL-17 and IL-23 in paediatric systemic lupus erythematosus. *Lupus*.

[21] Tabarkiewicz J., Pogoda K., Karczmarczyk A., Pozarowski P., Giannopoulos K. (2015). The role of IL-17 and Th17 lymphocytes in autoimmune diseases. *Archivum Immunologiae et Therapiae Experimentalis*.

[22] Jeon C., Sekhon S., Yan D., Afifi L., Nakamura M., Bhutani T. (2017). Monoclonal antibodies inhibiting IL-12, -23, and -17 for the treatment of psoriasis. *Human Vaccines & Immunotherapeutics*.

[23] Mease P. J. (2015). Inhibition of interleukin-17, interleukin-23 and the TH17 cell pathway in the treatment of psoriatic arthritis and psoriasis. *Current Opinion in Rheumatology*.

[24] Atwa M. A., Emara A. S., Youssef N., Bayoumy N. M. (2014). Serum concentration of IL-17, IL-23 and TNF-*α* among patients with chronic spontaneous urticaria: association with disease activity and autologous serum skin test. *Journal of the European Academy of Dermatology and Venereology*.

[25] Dańczak-Pazdrowska A., Kowalczyk M., Szramka-Pawlak B. (2012). Interleukin-17A and interleukin-23 in morphea. *Archives of Medical Science*.

[26] Plée J., le Jan S., Giustiniani J. (2016). Integrating longitudinal serum IL-17 and IL-23 follow-up, along with autoantibodies variation, contributes to predict bullous pemphigoid outcome. *Scientific Reports*.

[27] Pouralibaba F., Babaloo Z., Pakdel F., Abdollahian T., Pourzare S. (2012). Elevated levels of interleukin-23 in sera of patients with pemphigus vulgaris. *Iranian Journal of Immunology*.

[28] Xue J., Su W., Chen Z., Ke Y., du X., Zhou Q. (2014). Overexpression of interleukin-23 and interleukin-17 in the lesion of pemphigus vulgaris: a preliminary study. *Mediators of Inflammation*.

[29] Ben Jmaa M., Abida O., Fakhfakh R. (2018). Involvement of the IL23/Th17 pathway in the pathogenesis of Tunisian pemphigus foliaceus. *Mediators of Inflammation*.

[30] Vaccaro M., Cannavò S. P., Imbesi S. (2015). Increased serum levels of interleukin-23 circulating in patients with non-segmental generalized vitiligo. *International Journal of Dermatology*.

[31] Wang H., Luo Z., Lei L. (2013). Interaction between oral lichen planus and chronic periodontitis with Th17-associated cytokines in serum. *Inflammation*.

[32] Ibrahim A., ZT L., AA N., GS B. (2015). Interleukin 23 and interleukin17 in psoriasis, atopic dermatitis and lichen planus: a serological study. *Zagazig University Medical Journal*.

[33] Chen J., Feng J., Chen X. (2013). Immunoexpression of interleukin-22 and interleukin-23 in oral and cutaneous lichen planus lesions: a preliminary study. *Mediators of Inflammation*.

[34] Mardani M., Torabi Ardakani S., Dastgheib L., Hamidizadeh N. (2020). Serum levels of IL-22 in patients with oral lichen planus and cutaneous lichen planus. *Journal of Dentistry*.

[35] Lu R., Zeng X., Han Q. (2014). Overexpression and selectively regulatory roles of IL-23/IL-17 axis in the lesions of oral lichen planus. *Mediators of Inflammation*.

[36] Gaffen S. L., Jain R., Garg A. V., Cua D. J. (2014). The IL-23-IL-17 immune axis: from mechanisms to therapeutic testing. *Nature Reviews. Immunology*.

[37] Tonel G., Conrad C., Laggner U. (2010). Cutting edge: a critical functional role for IL-23 in psoriasis. *Journal of Immunology*.

[38] Kimball A. B., Gordon K. B., Langley R. G. (2008). Safety and efficacy of ABT-874, a fully human interleukin 12/23 monoclonal antibody, in the treatment of moderate to severe chronic plaque psoriasis: results of a randomized, placebo-controlled, phase 2 trial. *Archives of Dermatology*.

[39] Kasper L. H., Everitt D., Leist T. P. (2006). A phase I trial of an interleukin-12/23 monoclonal antibody in relapsing multiple sclerosis. *Current Medical Research and Opinion*.

[40] van Vollenhoven R. F., Hahn B. H., Tsokos G. C. (2018). Efficacy and safety of ustekinumab, an IL-12 and IL-23 inhibitor, in patients with active systemic lupus erythematosus: results of a multicentre, double-blind, phase 2, randomised, controlled study. *Lancet*.

[41] Gooderham M. J., Papp K. A., Lynde C. W. (2018). Shifting the focus - the primary role of IL-23 in psoriasis and other inflammatory disorders. *Journal of the European Academy of Dermatology and Venereology*.

[42] Chan T. C., Hawkes J. E., Krueger J. G. (2018). Interleukin 23 in the skin: role in psoriasis pathogenesis and selective interleukin 23 blockade as treatment. *Therapeutic Advances in Chronic Disease*.

[43] Kim J., Krueger J. G. (2017). Highly effective new treatments for psoriasis target the IL-23/type 17 T cell autoimmune axis. *Annual Review of Medicine*.

